# Macrophages and Stem Cells—Two to Tango for Tissue Repair?

**DOI:** 10.3390/biom11050697

**Published:** 2021-05-06

**Authors:** Emilia Manole, Cristina Niculite, Ioana Maria Lambrescu, Gisela Gaina, Octavian Ioghen, Laura Cristina Ceafalan, Mihail Eugen Hinescu

**Affiliations:** 1 Cell Biology, Neurosciences and Experimental Myology Laboratory, Victor Babeș Institute of Pathology, 050096 Bucharest, Romania; emilia_manole@yahoo.com (E.M.); cristina.niculite@umfcd.ro (C.N.); ioana.lambrescu@umfcd.ro (I.M.L.); giselagaina@yahoo.com (G.G.); octavian.ioghen@umfcd.ro (O.I.); mihail.hinescu@umfcd.ro (M.E.H.); 2 Pathology Department, Colentina Clinical Hospital, 020125 Bucharest, Romania; 3 Department of Cellular and Molecular Biology and Histology, Carol Davila University of Medicine and Pharmacy, 050474 Bucharest, Romania; 4 Department of Biochemistry and Molecular Biology, University of Bucharest, 030018 Bucharest, Romania; 5 Department of Clinical Neurosciences, Carol Davila University of Medicine and Pharmacy, 050474 Bucharest, Romania

**Keywords:** macrophage, stem cell, stem cell niche, tissue regeneration

## Abstract

Macrophages (MCs) are present in all tissues, not only supporting homeostasis, but also playing an important role in organogenesis, post-injury regeneration, and diseases. They are a heterogeneous cell population due to their origin, tissue specificity, and polarization in response to aggression factors, depending on environmental cues. Thus, as pro-inflammatory M1 phagocytic MCs, they contribute to tissue damage and even fibrosis, but the anti-inflammatory M2 phenotype participates in repairing processes and wound healing through a molecular interplay with most cells in adult stem cell niches. In this review, we emphasize MC phenotypic heterogeneity in health and disease, highlighting their systemic and systematic contribution to tissue homeostasis and repair. Unraveling the intervention of both resident and migrated MCs on the behavior of stem cells and the regulation of the stem cell niche is crucial for opening new perspectives for novel therapeutic strategies in different diseases.

## 1. Introduction

Aside from their contribution to innate and adaptive immunity, macrophages (MCs) orchestrate a series of complex phenomena, such as homeostasis, development, and even neoplastic progression [1,2]. Increasing evidence supports the role of MCs in the healing process of various injured tissues. The disruption of MC function has been suggested to account, at least partially, for impaired regeneration and scar formation. Thus, in recent years, they have been regarded as potentially important therapeutic targets [3,4].

The healing process consists of three overlapping phases: inflammation, tissue regeneration, and tissue remodeling, along with angiogenesis and extracellular matrix (ECM) reorganization [5]. The molecular pathways that regulate each phase of the healing process were extensively studied in order to identify new targets for improving and accelerating the process [5]. MCs were reported to constantly participate in all phases. However, in some circumstances, MCs can also become detrimental to the healing process, due to the uncontrolled production of pro-inflammatory cytokines and cytotoxic radical species, as seen in several human diseases [6,7].

During the inflammatory stage, immediately after tissue injury, danger or damage-associated molecular patterns (DAMPs) bind to their associated pattern-recognition receptors and recruit immune cells [8]. One of the first DAMP proteins described is the high-mobility group box-1 protein (HMGB1), which has an important role in immune cell recruitment, acting as a chemoattractant for monocytes. It has also been reported as a reprogramming factor for MCs towards a pro-fibrotic phenotype [5,9,10]. The injured tissue also releases nuclear and mitochondrial nucleic acids, which may act as DAMPs, inducing an inflammatory response. These extracellular DNA fragments are phagocytized by MCs, where they stimulate the inflammasome, an intracellular multiprotein complex receptor of innate immunity [11,12]. In turn, the inflammasome upregulates pro-inflammatory cytokine production (IL-1β, IL-18, TGF-β), activating fibrotic signaling [13,14], and TNF-*α*, which promotes the recruitment of other inflammatory cells [15]. The inflammation is resolved through the intervention of specific mediators. A wide array of anti-inflammatory cytokines are produced by local MCs [15]. Among them, resolvins (RVs), bioactive lipid compounds, are upregulated by anti-inflammatory MCs. RVs act as inhibitors of neutrophil chemotaxis at the injury site [16,17].

However, through the production of matrix metalloproteinases (MMPs), MCs not only facilitate stem cell proliferation and growth, but can also promote fibrosis [18]. For example, during this phase, MMP-12, a major macrophage-secreted elastase, induces a fibrotic phenotype, increasing the expression of some pro-fibrotic markers in injured skin and the heart [19]. The pro-fibrotic phenotype is also induced by interleukin-4 receptor alpha (IL-4R α) through modulation of collagen fibril deposition [20,21]. IL-4R α stimulates MC activation and maturation, playing an important role in tissue repair. Under the influence of activated MCs, fibroblasts produce lysyl hydroxylase 2 in the event of fibrosis [22].

Adult stem cells reside in a specialized microenvironment, called the stem cell niche, which provides integration of intrinsic and extrinsic determinants, influencing the fate of stem cells. Although they are tissue-specific, the stem cell niches are defined by common features, both structural and functional. They consist of supporting stromal cells, various ECM proteins, and signaling events through direct molecular interactions or soluble molecules that control the activation of stem cells or maintain their quiescence and self-renewal (for review, see [23]).

Among other heterogeneous cell types that are part of the stem cell niche, MCs were frequently reported as important players in the cross-talk with stem/progenitor cells, contributing to tissue repair in various organs, such as skeletal muscle, heart, liver, kidney, and even mammary gland and testis [24,25]. MCs produce cytokines, such as interleukin-6 (IL-6), TNF-*α*, and PGE_2_, which were demonstrated to promote stem cell proliferation in injured skeletal muscle, while another set of cytokines and growth factors, such as interleukin-4 (IL-4) and insulin-like growth factor-1 (IGF-1), were shown to stimulate further differentiation and growth [15].

Tissue MCs consist of two classes with phenotypic differences, resident MCs, and infiltrating MCs derived from monocytes. The first type is present in normal tissues, while the second type is recruited by circulating monocytes after tissue injury. During organogenesis, tissue-resident MCs originate from the embryonic hematopoietic progenitors in the yolk sac and fetal liver, and from the definitive hematopoietic stem cells after birth [26,27].

Tissue-resident MCs are found in all mammalian organs as immune sentinels and scavengers, providing some homeostatic functions. In recent years, various studies on organ transplantation showed that MCs can self-maintain for years in transplanted organs, such as lungs [28,29,30,31] and the heart [32]. Tissue microenvironments, considered the MC niche, control the population size and determine their tissue-specific identity [33]. Their development and homeostasis require constant stimulation through trophic factors, such as interleukin- 34 (IL-34), colony stimulating factor 1 (CSF1/M-CSF), or CSF2 (or GM-CSF) [33]. While IL-34 seems to be important for maintaining the number of Langerhans cells and brain microglia, CSF1 is required for the proliferation of all other tissue-resident macrophages, which is dependent on CSF1 bioavailability. A recent study proposed a “territory model”, where contact inhibition may be responsible for their tissue distribution [33].

Moreover, plasticity is another hallmark of MCs, rendering them capable of changing their phenotype according to environmental stimuli [34]. Consequently, the MC population can be subdivided into classical activated (M1) and alternatively activated (M2). M1 MCs are involved in the secretion of pro-inflammatory cytokines (IL-6, IL-12, TNF-α) and the production of reactive oxygen species (ROS) and nitric oxide (NO). In contrast, secretion of high amounts of anti-inflammatory cytokines (IL-10 and TGF-β) and small amounts of pro-inflammatory cytokines (such as IL-12, TNF-α) is a characteristic of M2 MCs [34,35].

A very recent study showed that the polarization of MCs from the M1 to M2 phenotype can also be modulated by mesenchymal stem cell-derived extracellular vesicles (MSC-EVs). They reduce M1 polarization and/or promote M2 polarization in a variety of settings. The administration of MSC-EVs was proven to have a beneficial effect in various lung, heart, digestive system, kidney, and central nervous system (CNS) diseases. However, the molecular mechanisms for MC targeting and EV uptake by MCs are not completely resolved [36].

Recently, Sath et al. highlighted the existence of another MC atypical phenotype, critical for fibrosis, Ceacam1^+^Msr1^+^Ly6C^−^F4/80^−^Mac1^+^ monocytes, which was named segregated-nucleus-containing atypical monocytes (SatM). These macrophages share granulocyte characteristics and they are regulated by CCAAT/enhancer binding protein (C/EBPβ) [37].

The identification and elucidation of both structural and functional elements that build and regulate the stem cell niche in health and disease are crucial for developing therapeutic strategies for patients with different diseases.

This review focuses on the intervention of local or migrated MCs on the behavior of stem cells across various organs during homeostasis and tissue regeneration. We suggest that the delicate “dance” between stem cells and MCs participates in the complex process of tissue (re)generation in every organ, highlighting evidence that “MCs can be thought of as a dispersed homeostatic organ” [26].

## 2. “The Floor” for the MC-Stem Cell Tango

### 2.1. Bone Marrow

In the bone marrow, hematopoietic stem cell (HSC) niches are specific environments [38] that preserve their self-renewal and differentiation capacity into all blood cell types [39]. Active and quiescent HSCs, which are recruited when needed, and then return to dormancy once the depleted cell population has been replenished, occupy two separate niches within the bone marrow [40]: the endosteal niche, at the interface between compact bone and the bone marrow [41], which contains quiescent HSCs and is associated with low blood perfusion and hypoxia, and the perivascular niche, with a higher oxygen content, holding active HSCs [42,43,44,45].

Multiple reports have documented the importance of MCs in regulating the local stem cell niches [45,46].

The perivascular niche is located around the sinusoids in the bone marrow, and contains a specific subset of MCs that support maintenance and proliferation of mesenchymal stem cells (MSCs). Through their interaction with MSCs in the niche they regulate expression of retention molecules for HSCs in the bone marrow, such as CXCL12 (C-X-C motif chemokine ligand) [46,47].

**Figure 1:**
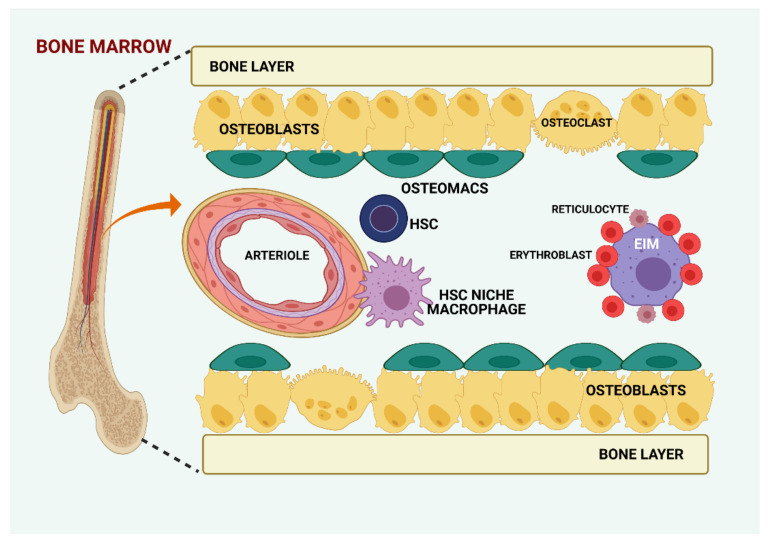
Resident macrophage subsets in bone marrow. There are three resident macrophage subsets in bone and bone marrow: erythroblastic island macrophages (EIM), which interact with erythroblasts and support them during erythropoiesis, hematopoietic stem cell (HSC) niche macrophages, and osteal macrophages (osteomacs). The perivascular niche contains a specific subset of macrophages that support maintenance and proliferation of mesenchymal stem cells and retain HSCs within the bone marrow. Osteomacs can be found immediately adjacent to osteoblasts and regulate bone formation and homeostasis. Created with BioRender.com (accessed on: 7 March 2021).

The endosteal niche contains a specific subset of tissue-resident MCs, termed osteomacs, which are involved in bone formation and homeostasis [48] (Figure 1). They are positive for F4/80, CD169, vascular cell adhesion molecule 1 (VCAM1), CD11b, CD68, CD115, and macrophage-3 antigen (MAC3), and are negative for tartrate-resistant acid phosphatase (TRAP) [41,49,50]. The human orthologs for CD169^+^VCAM1^+^ murine MCs were identified as CD15^-^CD163^+^VCAM1^+^CD169^+^ human bone marrow MCs [51]. It is not yet clear whether the murine MCs described by Winkler et al. [41] are different from the murine CD169^+^ MCs described by Chow et al. [49].

Furthermore, they regulate maintenance and proliferation of Nestin^+^ MSCs. These MSCs express a variety of factors for the retention of HSCs in the niche [41,52,53]. CD169^+^ MCs have been identified as the population of MCs that promote retention of HSCs in the endosteal niche. Studies showed that in vivo depletion of MCs, in either macrophage Fas-induced apoptosis (MaFIA) transgenic mice or by administration of clodronate-loaded liposomes into wild-type mice, led to marked reduction of endosteal osteoblasts and HSC mobilization into blood, mimicking the phenotype observed during granulocyte colony-stimulating factor (G-CSF) administration [41,54]. CD169^+^ MCs secrete oncostatin M (OSM), which in turn induces Nestin^+^ cells and possibly other MSCs, to express CXCL12 [49,55]. A rare population of MCs is positive for α-smooth muscle actin and cyclooxygenase-2, localized adjacent to HSCs in the bone marrow, which may protect HSCs from exhaustion by limiting the production of ROS under stress [56]. Another study reported the existence of a subset of MCs expressing the Duffy antigen receptor for chemokines (DARC; also known as ACKR1) that regulate HSC quiescence via TGFβ–SMAD3 signaling [57].

Apart from HSC niche macrophages and osteomacs, another subset of MCs is also present in the bone marrow, the erythroblastic island macrophages (EIM), which aid erythroblasts during the maturation process by secreting cytokines, phagocytizing extruded nuclei, and expressing erythropoietin [40] (Figure 1). 

HSC niche MCs respond directly to stimuli during bone marrow injury, infection, or systemic inflammation and translate these stress signals to modulate HSC niche dynamics to achieve emergency hematopoiesis, a process which expedites myeloid cell production [40,46]. G-CSF produced by endothelial cells is a factor mediating HSC mobilization to peripheral blood following transplantation, as well as during infection [45,58,59]. To promote HSC mobilization, G-CSF acts on different cells from the HSC niche, including MCs [48]. Administration of G-CSF reduces the expression of retention molecules, such as *Cxcl12*, osteopontin, *Kitl*, *Angpt1* (angiopoietin 1), and *Vcam1* in bone marrow MSCs [60] and *Cxcl12* in osteolineage cells [61]. G-CSF can also affect the levels of CXCL12 in the endosteal niche through depletion of osteomacs [41]. Several studies suggest that resident MCs persist after myeloablative treatment (through radiation [62,63] or chemotherapy [64]) and are a key element in the re-establishment of the HSC niche following bone marrow injury or post-transplantation [65]. The expression of VCAM-1 by radiation-resistant MCs contributes to the maintenance of HSCs in the niche [66]. CD234^+^ bone marrow MCs also promote HSC quiescence [57]. These observations suggest that bone marrow MCs play an important part in protecting the long-term HSC pool and in the recovery of quiescent HSC niches post-transplant or following systemic infection [65,67].

### 2.2. Liver

**Figure 2:**
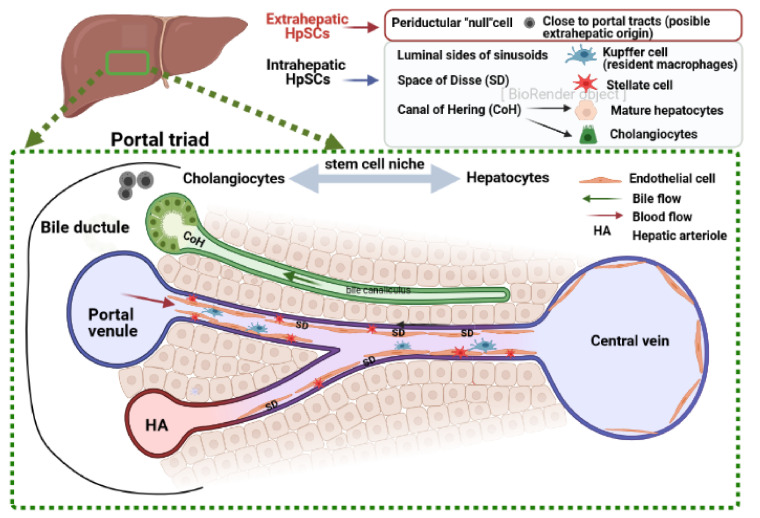
Schematic representation of various sources of hepatic stem cells with putative niches and their location within the liver. The portal triad consists of a portal venule, a bile ductule, and a hepatic arteriole. The blood in the portal venule and the hepatic arteriole flows between the hepatocytes to the central vein through sinusoids surrounded by sinusoidal endothelial cells. The bile secreted by hepatocytes is collected by a system of ducts. Four possible hepatic stem cell niches have been identified to date [71]. Three of them have intrahepatic localization: in the epithelium of the canals of Hering, space of Disse, and intralobular bile ducts. With extrahepatic origin, periductular “null” cells have been identified as a proximal niche [72]. Created with BioRender.com (accessed on: 7 March 2021).

Recent evidence indicates that, in the liver, there are multiple stem cell niches containing hepatic stem/progenitor cells (HpSCs), located in different anatomical regions. In adult tissue, the hepatic progenitor cell niches are present in the epithelium of the canals of Hering (CoH) [68] and also within the Disse space (DS) [69]. Recently, a new population of proliferating and self-renewing cells adjacent to the central vein in the liver lobule has been discovered [70]. It consists of unipotent Axin2^+^ hepatocyte progenitors bound to the endothelium, forming the central vein and replacing senescent polyploid hepatocytes in normal liver turnover [70]. However, the relationship of this niche with other stem/progenitor cells is not yet well defined (Figure 2).

The cellular elements of the niches include portal myofibroblasts, hepatic stellate cells (HStCs), cholangiocytes, hepatocytes, endothelial cells, pit cells, inflammatory cells, resident MCs (i.e., Kupffer cells (KCs)), and ECM as a scaffold. It is assumed that all these cells could interact and crosstalk with liver progenitor cells, influencing their proliferation and differentiation, either by direct contact or by the production of some humoral factors [73,74]. Moreover, it was shown that M-CSF is a key factor for the differentiation and proliferation of Kupffer cells in the liver [75].

The heterogeneous cell population of bone marrow mesenchymal stem cells (BM-MSCs) has been suggested to play a role in liver regeneration. Only a small (1–2% of BM-MSCs) subpopulation of BM-MSCs, known as multilineage-differentiating stress-enduring (Muse) cells, expressing pluripotency markers, such as SSEA-3, Nanog, Oct3/4, and Sox2, is able to differentiate into major liver components, including hepatocytes [76,77].

As for other organs, hepatic MCs have emerged as important players and key cellular components of the liver, with a role in the maintenance and stimulation of the hepatic progenitor niche. Hepatic MCs consist of resident liver KCs and bone marrow-derived recruited monocytes (BMDMs), which rapidly infiltrate the liver during injury and differentiate to MCs. Both populations of hepatic MCs play an important role in maintaining homeostasis of the liver, as well as in various liver diseases, being involved in inflammatory and anti-infectious processes. KCs are derived from fetal liver monocytic precursors that expand and maintain themselves through the entire life of the organism [78] by influencing liver progenitor cell invasion and phenotype through chemoattractant factors [79]. Recently, another liver-resident macrophage population located in the hepatic capsule has been described by Sierro F et al. [80]. These liver capsular MCs (LCMs), which arise from adult circulating monocytes and not from embryonic precursors, are distinct from KCs from a phenotypical and developmental point of view.

KCs and MCs can be distinguished based on their expression of cell surface markers. In mice, KCs are CD11b ^low^, F4/80^high^, and Clec4F^+^, while MCs are CD11b^+^, F4/80 ^int^, Ly6C^+^, and CSF1R^+^ [81,82,83]. LCMs are CD11b^+^F4/80^+^CD11c^+^MHC-II^+^CSF1R^+^ and are negative for Ly6C, Clec4F, and TIM4, which shows a difference from KCs and MCs [84].

The contribution of hepatic MCs to liver homeostasis is demonstrated by an intimate cross-talk with other cell types, such as HStCs, modulating their activation and viability by the release of cytokines and other factors, such as Wnt ligands (e.g., Wnt3a) or growth factors. Thus, hepatic MCs play an important role in liver fibrogenesis and fibrosis resolution [85]. Once the liver damage has occurred, the resident hepatic MCs interact with HStCs, the primary collagen-producing cells, which differentiate into proliferative, migratory, and contractile-activated myofibroblasts and secrete several profibrotic/pro-inflammatory factors [86]. Another signaling pathway present in the HpSC niche is Notch, which promotes the differentiation of hepatic progenitor cells into cholangiocytes via the ligand jagged 1, secreted by myofibroblasts [69].

Composition of the ECM has a special importance in the response of HpSCs to different stimuli [87]. MCs and HpSCs play a crucial role in the synthesis of ECM components [88] and ECM remodeling through the production of MMPs and their inhibitors [89]. The intervention of MMPs in the degradation of the collagen matrix, combined with the production of a laminin-rich niche, leads to the expansion of HpSCs, while the loss of the laminin-rich niche triggers the HpSCs differentiation into hepatocytes [90].

In addition to signals passing from niche cells to stem cells or progenitor cells, there may also be signals from the stem or progenitor cells to the niche [89] via the Hedgehog pathway. HpSCs can activate HStCs determining the release of liver matrix components responsible for normal liver regeneration [68,91]. Many challenges remain regarding the niche components in the liver and the crosstalk between them [73].

### 2.3. Intestine

The intestinal lumen is covered with a single-cell layer of epithelial tissue, organized into crypt-villus units composed of different cell types, including intestinal stem cells (ISCs) [92]. Besides its role in digestion, the mammalian intestinal tract is considered to be the main and largest immune organ of the body. It has an important role in managing host homeostasis and protection against pathogenic microorganisms [93].

Due to permanent exposure to microbial products and different dietary antigens, the intestinal epithelium is one of the most rapidly self-renewing tissues.

Intestinal stem cell niches are located at the base of the crypts. They are interspersed with Paneth cells (e.g., Lgr5^+ve^ cells) in the small intestine and Paneth-like cells in the colon or near position 4 within the intestinal crypt (e.g., DCAMKL-1 or Bmi1^+ve^ cells) [94].

They consist of the cellular microenvironment and the adjacent Paneth cells, epithelial cells, stromal cells, immune cells, smooth muscle cells, and fibroblasts, as well as ECM components (fibronectins, laminin isoforms, collagens, and glycosaminoglycans) and growth factors for the control of ISC proliferation and differentiation. The discovery of active Lgr5^+^ (ISCs marked by the leucine-rich repeat-containing G protein-coupled receptor 5) at the crypt base was a step forward in the field of ISC research [95]. These cells mark active cycling ISCs, maintain homeostasis, and have the capacity of long-term self-renewal and differentiation into different epithelial cell types. Another subpopulation of quiescent ISCs is phenotypically characterized by the expression of Bmi1 [96], Hopx, mTERT, and Lrig1 [97]. All these cell populations act as stem cells during the regeneration of the intestinal epithelium. Several signaling pathways are involved in communication between the ISCs and their niches, such as the Wnt, Notch signaling, TGF-β, bone morphogenic protein (BMP), and Hedgehog pathways. If these pathways are disrupted, the niche is affected, which leads to an increased risk of intestinal diseases [98].

During differentiation, the ISCs give rise to daughter cells within the transit-amplifying (TA) zone. Subsequently, TA cells will proliferate and migrate upwards to the crypto-villus junction. At the level of the crypt-villus junction, TA cells will be terminally differentiated into different cell types, such as tuft cells, goblet cells, enteroendocrine cells, and enterocytes. Paneth cells migrate downward to the stem cell niche zone; this renewal process lasts 4–7 days [99]. This rapid self-renewal of the mammalian intestinal epithelium requires a tightly regulated balance between proliferation and differentiation. With this constant need for epithelial renewal, as well as maintaining mucosal homeostasis under the constant action of the microbiota, the intestinal mucosa contains the largest pool of MCs in the body [100].

The most important MC population resides in the lamina propria. Given the need for periodic regeneration of the intestinal epithelium, the supply of the MC pool is based on the replenishment by circulating Ly6C^+^ monocytes derived from HSCs in the bone marrow. Several studies have shown that the intestine also harbors self-maintaining MCs located within the intestinal wall. These MCs are intimately associated with surrounding cells, implying that they receive information from the macrophage niche [101]. It has recently been suggested that MCs functionally adapt to the niche in which they are located. It involves a combination of trophic factors, signaling molecules and physical scaffolds present in a specific tissue, able to imprint the precise function and phenotype on MCs [33].

However, intestinal mucosal damage due to inflammatory conditions determines MC accumulation. Recently, MCs have been identified as sources for Wnt factors [102], as targets of Wnt signals affecting their polarization, and as key components of ISC crypts [103].

The macrophage-derived Wnt ligands seem to modulate mucosal regeneration, while their persistence can lead to fibrosis. It is known that the Wnt signaling pathway is implicated in tissue homeostasis. However, rapid regeneration of the intestinal mucosa, which involves a high rate of cell proliferation/differentiation and a collaboration of several signaling pathways, is required. Thus, homeostasis depends on the balance of the Wnt signaling pathway, along with Notch and Hedgehog pathways [104].

### 2.4. Central Nervous System

Three main stem cell reservoir zones were described in the mammalian brain: the subgranular zone in the dentate gyrus of the hippocampus, the subventricular zone around the lateral ventricles, and the central canal of the spinal cord, which has so far been poorly investigated [105,106,107].

The neural stem cell (NSC) niche regulates the capacity of neural stem cells to remain dormant or activate and differentiate when necessary [108].

The niches comprise several cell types and the molecules they produce [109,110]. The subventricular niche includes neural stem cells, neuroblasts, astrocytes, ependymal cells, endothelial cells, and microglia [109]. The subgranular zone niche encompasses NSCs surrounded by intermediate progenitors, astroglial cells, microglia, and mature granule cells [110,111].

NSCs are undifferentiated cells that have the capacity of self-renewal and multipotency. They can migrate and participate in neurogenesis by giving rise to a variety of other cells from the CNS, either neurons or glial cells [112]. After migration and differentiation, they integrate in the preexisting circuits from the brain area, where they have migrated [109]. NSCs possess the unique capacity to stay dormant for very long periods and are activated mainly when tissue regeneration becomes necessary [112].

Microglia are the resident MCs in the CNS [109]. Even though their main role is the phagocytosis of pathogens, they also play other roles, such as pruning of dysfunctional synapses and regulation of synaptic plasticity [109]. Furthermore, they are also involved in neurogenesis as part of the stem cell niches [109].

An increasing amount of evidence shows that a continuous dialogue exists between microglia and NSCs, and that microglia play essential roles in the genesis of new neurons or glial cells during fetal brain development, but also in the adult brain [113,114]. The morphology and antigenicity of microglia in the adult brain is different in the stem cell reservoir zones from the other areas of the brain: they have a larger cell body, fewer and thicker processes, and lower expression of activated microglia markers, such as TREM2 and CD68 [113,115,116].

During embryogenesis, primitive c-kit^+^ erythromyeloid precursors found in the yolk sac represent the precursors of the definitive CNS microglial population. These cells develop into CD45^+^ c-kit^lo^ CX3CR1^-^ immature (A1) cells and mature into CD45^+^ c-kit^-^ CX3CR1^+^ (A2) cells, subsequently migrating towards the developing brain and invading it using MMPs (MMP-8 and MMP-9). The whole process is dependent on the transcription factor Pu.1 and Interferon Regulatory Factor 8 (Irf8) [117].

In the fetal brain, microglia regulate the number of progenitor neural cells [118]. Cunningham et al. found that microglia could limit cell production in the prenatal cortex by phagocytizing precursor cells, particularly in the final stages of cortical neurogenesis, in macaque monkeys and rats [114]. It is noteworthy that the phagocytosis of precursor neural cells during neurogenesis by microglia should not be regarded as a clean-up process. It was observed that microglia were phagocytizing viable, not apoptotic neurons, implying that microglia ultimately regulate the size of the cerebral cortex [114]. Moreover, microglia are thought to induce precursor neural cells to exit their proliferative mode, preparing them for differentiation [119].

**Figure 3:**
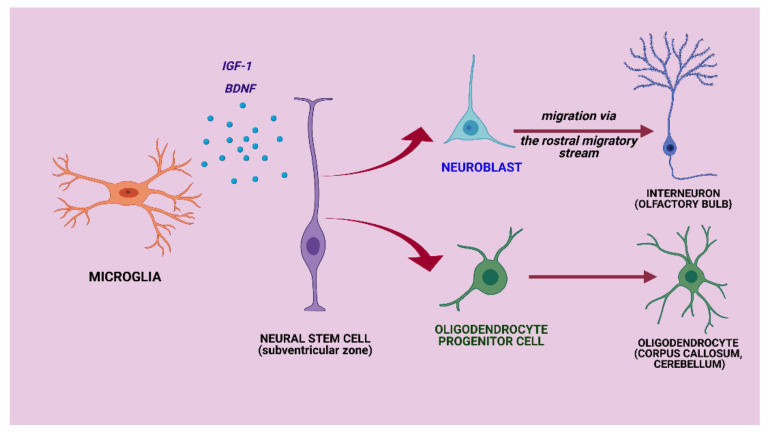
Neural stem cell niche. Under physiological conditions, the subventricular zone continuously provides neural progenitor cells that differentiate into neuroblasts, which migrate via the rostral migratory stream to the olfactory bulb, where they become interneurons [115,120]. Through IGF-1 and BDNF, microglia direct the neural stem cells towards the olfactory bulb, help them differentiate into interneurons, and integrate in the local circuitry [115,120]. The neural stem cell niche provides cells that are recruited in the corpus callosum and cerebellum and contribute to normal myelinogenesis by maintenance of the local oligodendrocyte population [124]. Insulin-like growth factor-1 (IGF-1), brain derived neurotrophic factor (BDNF). Created with BioRender.com (accessed on: 7 March 2021).

Regarding neurogenesis in the adult brain, microglia are considered to play a role in recruiting and spatially orienting the precursor neural cells towards their site of migration [115,120]. Microglia direct NSCs towards their site of migration, help them differentiate into a specific cell type, and help them integrate in the local circuitry [115]. For example, under physiological conditions, the subventricular zone continuously provides neural progenitor cells that differentiate into neuroblasts and migrate from the subventricular zone via the rostral migratory stream to the olfactory bulb, where they become interneurons [115,121,122]. Among the factors secreted by microglia that induce this effect are insulin-like growth factor 1 and brain-derived neurotrophic factor (BDNF) [123] (Figure 3).

Moreover, the NSC niche provides cells that are recruited in the corpus callosum and cerebellum and contribute to normal myelinogenesis by maintenance of the local oligodendrocyte population [124]. Under physiological conditions, the precursor neural cells from the subventricular zone mainly differentiate into neurons and only a small percentage to oligodendrocytes [125]. Under pathological conditions that imply demyelination, precursor neural cells are recruited, migrating towards oligodendrocytes, instead of neurons, with the aim of improving myelination in the injured areas [125]. It was demonstrated in an experimental model of cuprizone-induced demyelination that precursor neural cells are recruited, migrate towards the lesions of the corpus callosum, and differentiate into oligodendrocytes to aid in remyelination and confer neuroprotection, this process being orchestrated by microglial cells [126].

One signal by which microglia communicate with the neurons during neurogenesis is thought to be the chemokine fractalkine. It is expressed in healthy neurons and binds to a single receptor on microglia, CX3CR1 [127]. Experiments in mice and rats showed that a decrease in CX3CR1 expression leads to a decrease in the proliferation of hippocampal neural precursor cells and hippocampal neurogenesis [127]. In the aging brain, hippocampal neurogenesis is reduced compared to that in the young brain [127]. In the same series of experiments, exogenous fractalkine mitigated the decline in neurogenesis associated with aging [127]. 

Aging of the brain is associated with a shift in the microglia phenotype towards a more pronounced pro-inflammatory phenotype [128,129]. Among contributing factors, an increase of IL1β, IL16, and TNF-α production by microglia was documented [128,129]. Aging of the brain is a risk factor for Alzheimer disease, a neurodegenerative disease characterized by the accumulation of extracellular amyloid beta plaques [130]. It was demonstrated that mice with presenilin-1 (PS1) mutations in microglia, specific to familial Alzheimer disease, show impaired hippocampal neurogenesis and emotional function [130]. In further experiments, the same authors demonstrated that depletion of microglia restored the aforementioned deficits [130]. Based on these observations, Ortega Martinez et al. came up with the hypothesis that a cognitive decline in Alzheimer disease is caused by PS1-mutated microglia that fail to adequately orchestrate the neurogenesis in the hippocampal area [130].

### 2.5. Skeletal Muscle

**Figure 4:**
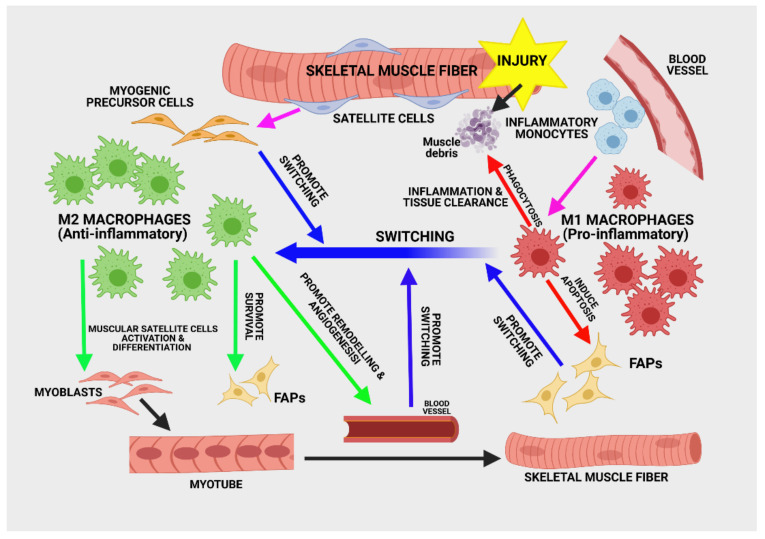
Skeletal muscle regeneration in acute muscle injury. Macrophages play a central role in both the inflammatory process and tissue remodeling. The inflammatory phase after injury is characterized by pro-inflammatory M1 macrophage accumulation, for tissue debris phagocytosis and myogenesis, by promoting satellite cell proliferation, regulating fibrosis by apoptosis of fibro-adipogenic progenitors (FAPs). After macrophages switch towards the anti-inflammatory M2 phenotype satellite cells are stimulated to generate myoblasts, myotubes, and myofibers. M2 macrophages also promote angiogenesis and FAPs survival. The M2 macrophage polarization is supported by myogenic precursor cells, endothelial cells, and FAPs. Created with BioRender.com (accessed on: 7 March 2021).

The resident stem cells of skeletal muscle are satellite cells (SCs). They occupy a specific niche, enclosed by the basal lamina of the mature muscle fiber. This complex environment consists of ECM and cellular elements, along with SCs, such as adult host myofibers, inflammatory cells, fibro-adipogenic progenitors (FAPs), and even endothelial cells. After muscle injury, SCs become activated, proliferate, and differentiate to functional skeletal muscle cells. The healing process develops through three successive steps: degeneration and inflammatory response, regeneration phase, and repair, along with angiogenesis and matrix remodeling (Figure 4) [131]. However, SC transplantation for muscle repair has not proved to have the expected therapeutic effects.

Multiple resident MC cell populations [132,133] and fibro-adipogenic precursor cells [134,135] were reported to support muscle stem/progenitor cell activation, and there are many mechanisms contributing to ensure tissue homeostasis and regeneration [136]. 

There are complex cell interactions within the muscle stem cell niche after injury. Ratnayake et al. [137] showed an interconnection between SCs and the innate immune system throughout the repair process. During the inflammatory stage, there is an influx of monocytes that differentiate to M1 CD68^+^ pro-inflammatory MCs when they reach the muscle interstitium, where they release pro-inflammatory cytokines, such as TNF-𝛼, IFN-𝛾, and IL-1𝛽 [138]. Along with migrated leukocytes, they remove necrotic debris and apoptotic cells. Thus, they participate in the inflammatory response, but, through trophic factor release, they also support SC activation, proliferation, and angiogenesis. After phagocytosis ends, MCs suffer a phenotypic switch and become anti-inflammatory M2 MCs (Figure 4). The anti-inflammatory environment is crucial for promoting and further supporting tissue regeneration [138,139,140]. This conversion is driven by cytokines, such as IL-4, IL-10, and IL-13. Efferocytosis, cellular metabolism, and signaling pathways are involved here as well, promoting stem cell differentiation, angiogenesis, and ECM rebuilding. In their recent review, Chazaud et al. [131] suggested that, rather than fighting it, inflammation should be allowed to self-resolve, as it favors tissue recovery.

Ratnayake et al. [137] further showed that there is a specific subset of MCs within the injury site, creating a transient niche for stem cell proliferation. This MC subset, named “dwelling MCs”, secretes proliferative signals (mitogenic stimuli), such as the cytokine nicotinamide phosphoribosyltransferase (NAMPT, known as visfatin or PBEF in humans), which acts through the C-C motif chemokine receptor type 5 (Ccr5) present in muscle stem cells, modulating the repair process. The results of this research demonstrated that macrophage-derived niche signals for muscle stem cells could be utilized in therapeutic purposes for skeletal muscle disorders.

In resting skeletal muscle, resident MCs are located mainly in the perimysium and epimysium [141]. Upon a severe injury, more MCs differentiate from the recruited monocytes, migrating to the site of lesion [142]. A correlation between the severity of the injury and the macrophage response during and post-inflammation has not been investigated. However, there are multiple studies reporting that anti-inflammatory/restorative MCs act as supportive cells for myogenic progenitor cell differentiation and fusion, and are required for efficient muscle regeneration [132,133,143].

A model of muscle injury is progressive ischemia due to a peripheral artery disease. In a mouse model with unilateral femoral artery excision, the animals were treated with a macrophage/adipose-derived stem cell (ASC) mix injected into ischemic gastrocnemius muscle. The study reported an accelerated recovery of muscle function and restoration of normal architecture, reduced inflammation, and improved perfusion of the regenerating ischemic limb [144]. It is known that adipose-derived stem cells/conditioned MCs have an important role in immunomodulation and tissue regeneration [145]. Moreover, upregulation of CD206 revealed that adipose-derived stem cells are very important modulators of bone marrow MC functional status [144]. 

An in vitro study using a coculture system of RAW264.7, MCs and ASCs under hypoxic conditions showed the induction of MCs to M2 phenotype in an ASC paracrine way [146]. Allogenic ASCs can enhance MC recruitment, driving their switch to M2 phenotype through the hypoxia inducible factor-1α (HIF-1α)/IL-10 pathway. Thus, the ASC-derived IL-10, mediated by HIF-1α, activated the signal transducer and activator of the transcription 3 (STAT3)/Arginase (Arg-1) pathway. The in vivo experiment on a limb hypoxia mouse model of transplanted ASCs revealed an immunomodulatory action by recruiting and enhancing the M2 MCs and increasing blood flow reperfusion of the injured limb.

Another interesting cooperation during muscle regeneration, involving MCs and FAPs [147], showed that a subpopulation of FAPs, which express surface marker VCAM1, activated by muscle injury and displaying a pro-fibrotic profile, regulate macrophage inflammatory response, contributing to muscle regeneration [148]. Other studies revealed an interrelation between MCs and FAPs in disease state, such as Duchenne muscular dystrophy (DMD) or animal models, such as *mdx* mice. In this latter case, after pharmacological switching of their phenotype, MCs were found to induce TGF-β released by FAPs, consequently reducing fibrosis [149,150].

Recent data showed that MCs directly induced SC activation through ADAMTS1 secretion [151], a metalloproteinase which suppresses the quiescence regulator NOTCH1. Another factor secreted by MCs is GDF3, an inducer of myoblast fusion and myotube formation [152]. After injury, MCs secrete glutamine, activating mTOR signaling in SCs, which further promotes their proliferation and differentiation [153]. 

Data coming from in vitro 3D culture models proved that anti-inflammatory MCs stimulate myogenesis-angiogenesis through OSM, contributing significantly to muscle regeneration [154]. A 3D model incorporating BMDMs, following a muscle injury, showed almost complete tissue repair [155]. The presence of BMDMs supported SC proliferation and differentiation, while their absence led to a SC pool depletion and progressive muscle degeneration. Moreover, BMDMs limited myofiber apoptosis and attenuated the pro-inflammatory environment. Similar results were obtained with human blood-derived MCs. An in vivo study [155] showed that the BMDMs incorporated within engineered muscle implants enhanced blood vessel ingrowth, cell survival, and muscle contractile function. BMDMs were shown to have an anti-apoptotic effect on injured muscle via cell-contact-mediated mechanisms, but also by macrophage paracrine action, downregulating TNF-α and IL-1β expression [156,157,158,159,160,161]. 

Qiu et al. [162] demonstrated in an in vivo rat volumetric muscle loss model that MSCs and decellularized ECM scaffold contributed to the promotion of skeletal muscle regeneration by synergistically triggering MC polarization toward the M2 phenotype, while suppressing the M1 phenotype. ECM scaffold implantation had some favorable effects, such as stimulation of perivascular stem cell mobilization, enhancing the presence of myogenic progenitor cells and myotube formation [163].

The complex interaction mechanisms between different cell types during skeletal muscle regeneration are not yet fully known. Ceafalan et al. [164] revealed, in an in vivo model of skeletal muscle injury (mouse model of crush injury), a new type of interaction between MCs and myogenic cells by direct heterocellular surface apposition over large areas and long linear distances throughout all stages of myogenesis. Thus, co-transplantation of myoblasts along with activated MCs could open new perspectives in therapeutic approaches post-injury.

### 2.6. Cardiac Muscle

As in skeletal muscle, MCs play an important role in cardiac muscle regeneration, especially after myocardial infarction, following a similar series of events. 

With their special plasticity in response to environmental signals, after myocardial infarction, MCs start as pro-inflammatory M1 MCs, present in the acute phase [165,166], and M2 MCs, immunosuppressive and reparative, present during regeneration and healing.

However, as the cardiac muscle stem cell niche is still a more elusive subject, the interplay between MCs and stem cells was explored in the special context of adipose tissue (AT)-derived MSCs. Some research reports showed that AT-MSCs can switch MCs from an inflammatory to an anti-inflammatory phenotype, contributing to tissue healing, especially under pathological conditions, such as myocardial infarction [167,168,169,170,171,172].

Adutler-Lieber et al. showed that the mediators involved in this process are IL-10, VEGF, IL-6, and IL-13 [145]. Cardiac AT-MSCs increased IL-10 secretion by MCs, suppressing inflammatory cytokine secretion, such as IL-1 α, TNF-α, IL-17, and IFN-ɣ by M1 MCs, and stimulated M2 MC phenotype activation, with a cardioprotective role. As a result, AT-MSCs significantly modified MC cytokine secretion and increased the release of anti-inflammatory and angiogenic cytokines, such as IL-10 and VEGF. The authors revealed that the interaction between AT-MSCs and MCs is bidirectional, MCs enhancing AT-MSCs secretion of M2 MCs inducers, such as IL-4 and IL-13. Moreover, AT-MSCs decreased MC phagocytic capacity. Although VEGF was secreted in large amounts by AT-MSCs, but not by MCs, in co-culture, it was shown that MCs stimulated AT-MSCs, increasing VEGF secretion. Another observation was that IL-6 and IL-13 are connected. Thus, incubating MCs with IL-6 resulted in an increased IL-13 secretion. IL-13, an anti-inflammatory cytokine, promoted M2 MC polarization. 

In cardiac regeneration after an injury, a fibrotic response is initiated, which leads to the formation of a post-injury scar. One of the MC roles is that of mediating the fibrotic response by activating cardiac fibroblasts. MCs can enhance the survival and activation of myofibroblasts by releasing TGF-β and fibroblast growth factors, indirectly influencing the scar formation [173]. Using unbiased transcriptomics, Simões et al. have shown an upregulation of collagen proximal to the injury site on in vivo zebrafish and mouse models with heart injury. They modulated the collagen formation with a transfer of MCs genetically depleted of collagen 4a3 binding protein (col4a3bpa) and its cognate collagen 4a1 (col4a1) and observed a significant reduction of pericardiac scar formation post-cryoinjury [174].

### 2.7. Mammary Gland

Mammary stem cells (MaSCs) are sustained by a specific microenvironment that constitutes the MaSC niche. The cellular repertoire of the human mammary gland is composed of basal and luminal cells that are derived from MaSCs [175,176]. It appears that luminal and myoepithelial cells, as well as the ECM and surrounding stromal cells, such as fibroblasts and adipose cells, play an important role in the regulation of the MaSC niche [177]. Moreover, aside from their contribution to the innate and adaptive immunity, local MCs orchestrate a series of complex phenomena, such as homeostasis, development, and neoplastic progression [1,178].

During puberty, the mammary gland undergoes a series of changes due to the hormonal context. The result of this process is the formation of the ductal tree orchestrated by terminal endbuds (TEBs) [179]. The MCs have been highlighted based on F4/80 expression along the neck of the TEBs during ductal elongation [180]. 

The study of Chakrabarti et al. reported the implication of the delta-like 1 (Dll1) Notch ligand in the crosstalk between MCs and MaSCs. The authors demonstrated that MCs are an essential component of the MaSC niche. Thus, it was observed that, in Dll1^cKO^ mammary glands in different development stages, the F4/80^+^ MC population was reduced [178]. Furthermore, mouse MaSCs produced Dll1, activating Notch signaling in MCs, which resulted in the production of Wnt ligands. Consequently, the endpoint of all these complex interactions is the proliferation of the MaSC population [178,181]. Other studies also demonstrated that the phenotype of the Dll1^cKO^ mammary gland, with reduced ductal elongation and branching, was similar to CSF1 knockout mice with MC deficiency [182].

### 2.8. Testis MCs in “The Spermatogonial Niche”

A sizeable percentage of the somatic cells found in the testis consist of tissue-resident MCs [183]. In addition to their immune function, testicular MCs (TMCs) proved to be more versatile than expected, taking part in tissue development, regulation of vascularization during embryonic growth, regeneration, and homeostasis [184,185]. Years of accumulated evidence indicate that TMCs play a pivotal role during development and adulthood [185,186] by maintaining the subtle balance between tolerance and reactivity [186], with emphasis on steroidogenesis and differentiation of spermatogonia [183,187].

**Figure 5:**
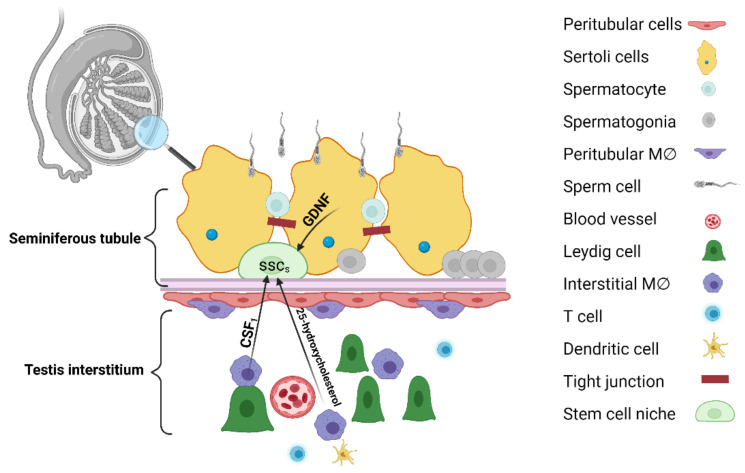
Overview of the testicular stem cell niche. The interstitial region is composed mainly of vascular structures, Leydig cells that produce testosterone, and macrophages. The spermatogonial stem cell (SSC) niche is located in the proximity of the basement membrane that separates the interstitium from the peritubular area. Adjacent Sertoli cells found in the seminiferous tubules form tight junctions that participate in the formation of the blood-testis barrier. In the testis, two populations of macrophages have been described: those that lie on the surface of the seminiferous tubules in the proximity of the peritubular myoid cells, comprising the peritubular population, and those located in the interstitium, intimately associated with Leydig cells. Colony stimulating factor 1 (CSF1), glial cell line-derived neurotrophic factor (GDNF), macrophage (MΦ). Created with BioRender.com (accessed on: 7 March 2021).

Depending on the location of MCs in the testis, two populations have been described: those that lie on the surface of the seminiferous tubules in the proximity of the spermatogonial stem cells (SSCs), comprising the peritubular population, and those located in the interstitium, intimately associated with Leydig cells [188] (Figure 5).

Peritubular MCs were thought to originate from circulating bone marrow-derived monocytes, while interstitial MCs differentiate from fetal yolk-sac-derived cells before being replaced by bone marrow-derived MCs after birth [184,188]. In contrast, Lokka et al. published an interesting study that examines the developmental origin and turnover of tissue-resident MCs in the testis. The authors indicate that fetal liver-derived precursors are an important source of interstitial MCs, while embryonic precursors generate peritubular MCs. According to their findings, even after systemic MC depletion, bone marrow-derived monocytes do not significantly contribute to the replenishment of the testicular MC pool [183]. Under physiological conditions, the in-silico fate mapping analyses tentatively classify fetal-derived CD206^-^MHCII^-^ macrophages as precursor cells that give rise to both interstitial CD206^+^MHCII- and peritubular CD206^-^MHC II^+^ macrophages. Locally proliferating CD206^+^MHCII^-^ cells possibly differentiate into CD206^-^MHCII^+^ cells when repopulating an empty testicular niche postnatally, and then take their peritubular role [183].

During steady-state conditions, the SSC population is located within the basal compartment of the seminiferous tubules. Spermatogenesis is the complete process of male germ cell development from stem cell pool to spermatozoa [189]. In fact, all the regulating mechanisms maintaining the balance between self-renewal and differentiation represent the stem cell niche. Sertoli cells are an important component that regulates the SSC niche via the glial cell line-derived neurotrophic factor (GDNF), the ligand for the GFRα1 receptor complex [190]. In addition, TMCs, peritubular myoid cells that communicate with Sertoli cells, the basement membrane, and differentiating germ cells, along with steroidogenic Leydig cells, all compete in the dynamics and preservation of the SSC niche [191]. The endothelium with its vascular-associated MCs and perivascular smooth muscle cells are also crucial components [192].

The testis is one of the immune-privileged sites in the mammalian body [193]. Tolerance to neo-antigens expressed on post-pubertal germ cells is orchestrated by a multitude of factors, from the physical blood–testis barrier to immune modulators, some of which are provided by local MCs [194]. Hence, the increased MC expression of CD163 and IL-10, corroborated with a low expression of pro-inflammatory cytokines, plays an important role in supporting an immune-privileged background [188,195].

The role of local MCs can be explained by their interactions with different components. The intercytoplasmic digitations formed between MCs and Leydig cells suggest their involvement in steroid biogenesis. Furthermore, TMCs produce 25-hydroxycholesterol, which is an intermediate compound within the testosterone biosynthetic pathway [196,197]. However, DeFalco et al. showed that, upon TMC ablation in mice, Leydig cells were mildly affected, with a testosterone concentration that did not fall below the mandatory limit for spermatogenesis [187].

CSF1 expressed in both interstitial and peritubular MCs could potentially impact spermatogonial behavior [187] via a colony-stimulating factor 1 receptor (CSF1R) expressed by the undifferentiated spermatogonial population. The interconnection between MCs and the SSCs and their involvement in spermatogenesis is also suggested by the chemotactic factor-encoding genes, such as *Ccl2, Ccl3, Ccl7, Csf1r, Cxcl2, Cxcl4*, and *Itgal*, which are enriched in both TMCs and the transcriptome of SSCs [198,199].

In order to differentiate, spermatogonia are dependent on the retinoic acid (RA) signaling pathway. Using immunofluorescence, DeFalco et al. demonstrated that two RA synthesis enzymes were expressed in TMCs. Hence, the MCs of the testis interstitium express ALDH1A2, while RDH10 was restricted mostly to peritubular and infrequently to interstitial MCs [187].

The depletion of TMCs, using the Cre-inducible diphteria toxin receptor (iDTR) method driven by *Cx3cr1*-Cre, was investigated by DeFalco et al. in order to better understand their extensive modulation in the testicular milieu. The diphtheria toxin injection revealed a significant overall reduction (>90%) in TMCs. However, this did not disrupt the short-term testicular function. Furthermore, a comparison between the control and MC-depleted testes showed similar numbers in Sertoli cells, with a maintained basic functionality of the Leydig cells, which were capable of sustaining a sufficient testosterone production for spermatogenesis [187].

### 2.9. Kidney

Studies on kidney mononuclear phagocytes began in recent years. In adult life, MCs can cause, prevent, and repair kidney damage [200,201]. There is scarce information about the origin of kidney MCs (KMCs) and their role during development. An increased number of MCs are found in diseased kidney, taking part in renal injury, inflammation, and fibrosis.

KMCs are very heterogeneous cells, with clear phenotypic characteristics, depending on various stimuli. As in other tissues, anti-inflammatory MCs mediate kidney regeneration [201]. After tissue injury, KMCs become polarized, with two subsequent subpopulations, the classically activated M1 and alternatively activated M2 MCs. M2 MCs are further subdivided into M2a, wound healing, M2b and M2c, and regulatory MCs [202].

KMCs were shown to express F4/80 [203,204,205,206]. These mononuclear phagocytes have an age-dependent developmental heterogeneity. The kidneys of newborn mice comprise a population of embryonic-derived MHCII^neg^F4/80^hi^CD11b^low^ MCs, expressing T cell Ig and mucin domain containing 4 (TIM-4) and MER receptor tyrosine kinase (MERTK). A few weeks after birth, these MCs are replaced by phenotypically similar cells that express MHCII but lack TIM-4 and MERTK. In acute kidney injury, MHCII^neg^F4/80^hi^ cells reappear in adult kidneys, possibly as a response to prostaglandin E2 and MHCII downregulation [207].

A genetic study revealed a role of MCs in nephron progenitor cell clearance in the early stages of mouse kidney development [208]. During renal organogenesis, most MCs are perivascular and express F4/80 and CD206. It is known that MCs support the kidney and its vasculature growth and development [209,210]. In vivo experiments, using genetically modified mice lacking MCs, showed that MCs are needed to clear away misplaced kidney cells and later interact with the growing blood vessel cells helping the vascular shaping network [208]. 

**Figure 6:**
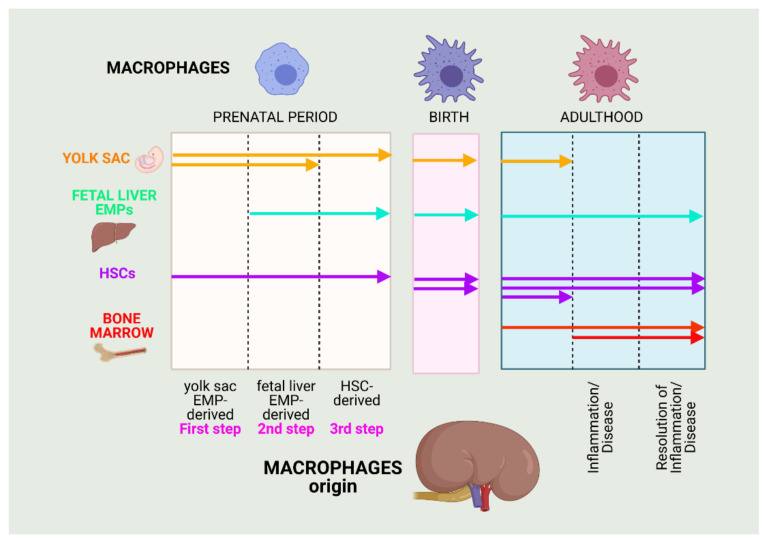
Macrophage origins in the kidney. MC sources in mice change throughout their development: in the prenatal period, at birth, and during adulthood. Thus, there are three sources in the embryo: yolk sac-derived erythro-myeloid progenitors (EMPs), fetal liver EMPs, and hematopoietic stem cells (HSCs). They constitute resident MC populations. Infiltrating MCs derive from bone marrow monocyte precursors and invade the kidney upon request, during an inflammatory process. Created with BioRender.com (accessed on: 7 March 2021).

Renal MCs have two origins, embryo-derived MCs (EMPs) and bone marrow-derived MCs. The EMPs are derived from yolk sac erythro-myeloid progenitors and fetal liver, plus HSC-derived MCs. They develop in parallel with kidney growth after birth and are mainly derived from fetal liver monocytes before birth. In adulthood, kidney MCs self-maintain, with contribution from peripheral monocytes. Munro and Hughes highlighted more studies, suggesting that EMPs enter the kidney to generate mature kidney MPs [211]. Circulating monocytes derived from bone marrow populate the healthy kidney at low levels, increasing after injury [212] (Figure 6).

Liu et al. showed that embryo-derived MCs are preferentially localized in the medulla/pelvis and have a higher capacity for scavenging immune complexes. Furthermore, these cells are more sensitive to immune challenge than bone marrow-derived MCs, having distinct glycolytic capacities. The epigenetic modifications of metabolic signaling pathways at the chromatin level can heighten the immune response of embryo-derived MCs by leading to functional modification or reprogramming of innate immune sensors and key inflammatory responses during embryonic development [213].

In human pathology, infiltration of MCs was found to be correlated with the severity of kidney injury, such as in glomerulonephritis [214,215,216,217,218]. Pro-inflammatory MCs play a role in the initiation and progression of kidney disease by secretion of pro-inflammatory mediators and interaction with kidney resident cells, but it was shown that MCs also contribute to the recovery phase of the disease, demonstrated in the ischemia/reperfusion injury model [219,220,221]. KMCs also contribute to renal fibrosis, which occurs when the repair process is insufficient or suppressed by inflammation. In recent years, the anti-fibrotic role of MCs has been demonstrated in obstructive nephropathy. Pro-inflammatory M1 MCs secrete inflammatory mediators, such as TNF-α and ROS, producing tissue inflammation and fibrosis. In contrast, anti-inflammatory M2 MCs release anti-inflammatory mediators, such as IL-10 and TGF-β, suppressing inflammation but promoting fibrosis [200].

## 3. Conclusions

Tissue regeneration requires the interaction and collaboration of several cell types in the stem cell niche. In this environment, MCs play a central role, coordinating and regulating the interrelation between these cells as they are, in turn, influenced by their activity. Thus, MCs can regulate inflammation, stem cell activation, proliferation, and differentiation, as well as vascularization and subsequent fibrosis, in an attempt to establish tissue homeostasis.

The knowledge about MC biology during the inflammatory response is growing and shows that they constantly adapt their gene expression to different phases of post-injury inflammation. It would be necessary to decipher the molecular mechanisms controlling MC biology in greater depth, as well as identify the common and specific processes and pathways that occur in various tissues and organs during inflammatory response. Many studies were conducted on skeletal muscle but could be translated and further investigated in other systems.

Although intensively studied, MCs still have much data to offer about their functions in different tissues, their longevity and distribution within tissue niche populations, and the influence of local factors on their activity.

This review highlights the fact that, aside from their well-known affiliation with the mononuclear phagocyte system, MCs also assemble a diffuse regenerative environment that ignites and promotes tissue repair. An array of signaling biomolecules bidirectionally conveys the “music”, resulting in a delicate but elegant metaphoric “dance” between MCs and stem cells. 

In the last decade, there has been an increasing amount of evidence that MCs arise as a promising therapeutic target for tissue regeneration. The concept of a MC transient niche for stem cell proliferation after injury, as proposed for the skeletal muscle, should be thoroughly investigated and translated for other organs in an attempt to support the hypothesis of a **MC diffuse regeneration system**.

## Data Availability

Not applicable.
